# Supplementary material for: Hand1 gene replacement with Hand2 reveals overlap in function with unique occurrence of omphalocele and heart defects
Source: Development. 2025 Oct 14;152(19):dev204963. doi: 10.1242/dev.204963 (PMC12587295; doi:10.1242/dev.204963)
Supplement: Supplementary information [file develop-152-204963-s1.pdf]

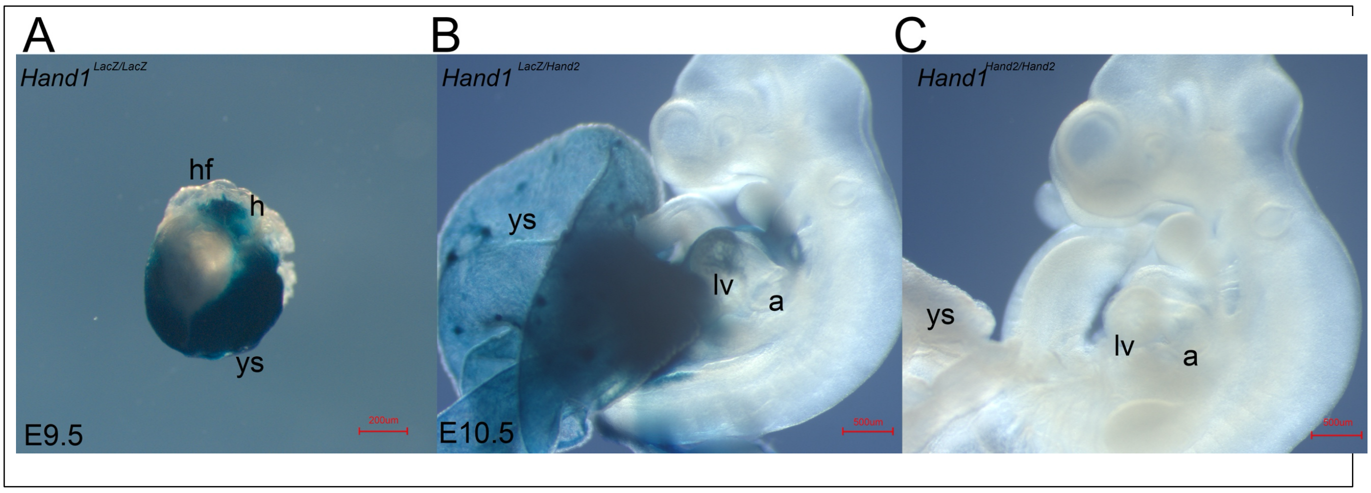

Fig. S1. *Hand^{LacZ}* intercross onto the *Hand1^{Hand2}* allele. A. an E9.5 *Hand1$^{LacZ/LacZ}$* embryo revealing severe extraembryonic defects within the yolks sac (ys) resulting in arrested heart development (h) and death, head fold (hf). **B.** an E10.5 *Hand1$^{LacZ/Hand2}$* demonstrating significant rescue of the systemic knockout phenotype. **C.** an E10.5 *Hand1$^{Hand2/Hand2}$* embryo, which lacks β-galactosidase staining, also exhibits rescue of the E9.5 systemic *Hand1* loss-of-function phenotype. Scale bar 200μm for panel A and 500μm for B and C.

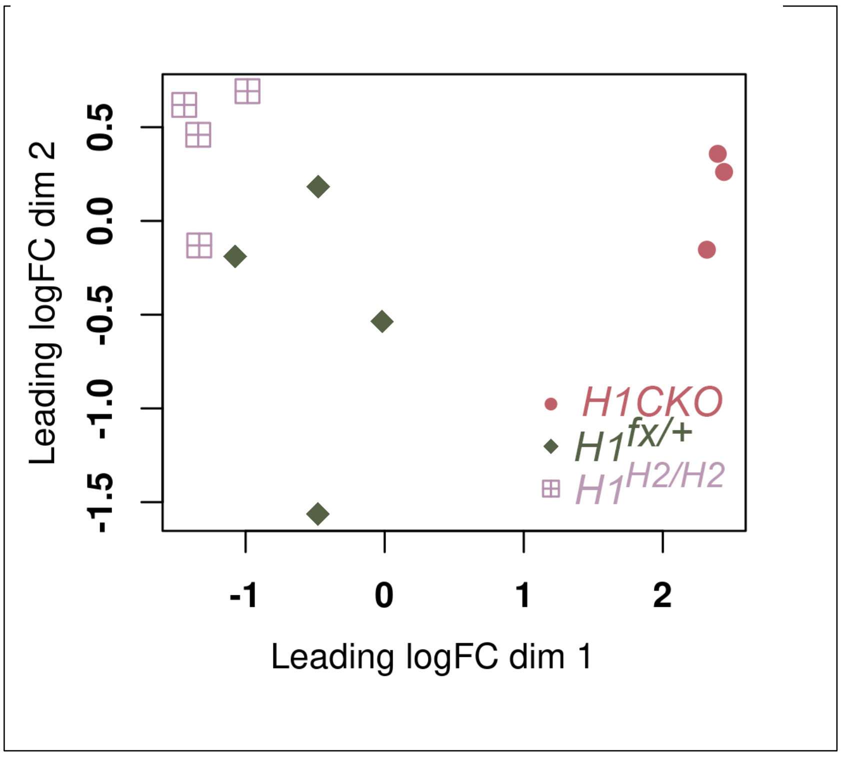

Fig. S2. Principal component analysis of yolk sac transcriptomic data. Comparison of *Control Hand1$^{fx/+}$* (*H1$^{fx/+}$*, green diamonds), *T-Cre;Hand1$^{fx/LacZ}$* (*H1CKO*, red circles) and *Hand1$^{Hand2/Hand2}$* (*H1$^{H2/H2}$*, purple squares) shows that *Control* and *H1$^{H2/H2}$* expression data are most similar where *H1CKO* expression data is more distinct. N=4 for both *Control* and *H1$^{H2/H2}$* yolk sac replicates and n=3 for *H1CKO* replicates as one of these yolk sacs was mis-genotyped and discarded from the study.

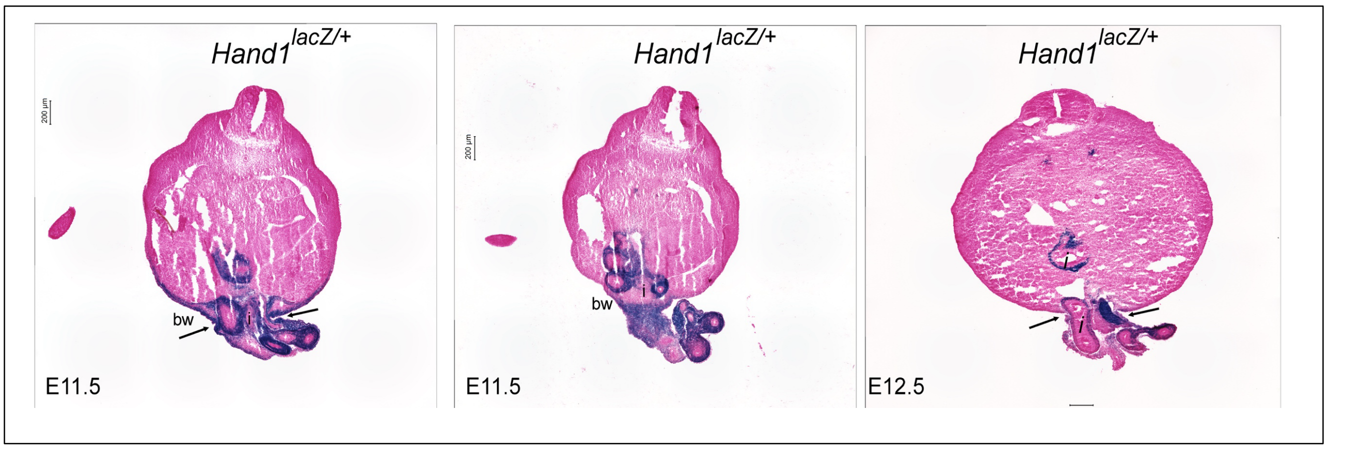

Fig. S3. β-galactosidase staining of E11.5 (left and center) and E12.5 (right) cryosections from *Hand1*$^{LacZ/+}$ embryos. LacZ staining within the ventral body wall (bw) surrounding the umbilical opening (arrows). Additional expression is observed within the developing smooth muscle layer of the intestines. E12.5 expression appears reduced but is still detectable indicating potential primary tissue sources of the observed omphalocele encountered in *Hand1*$^{Hand2/Hand2}$ mice**.**

Development • Supplementary information

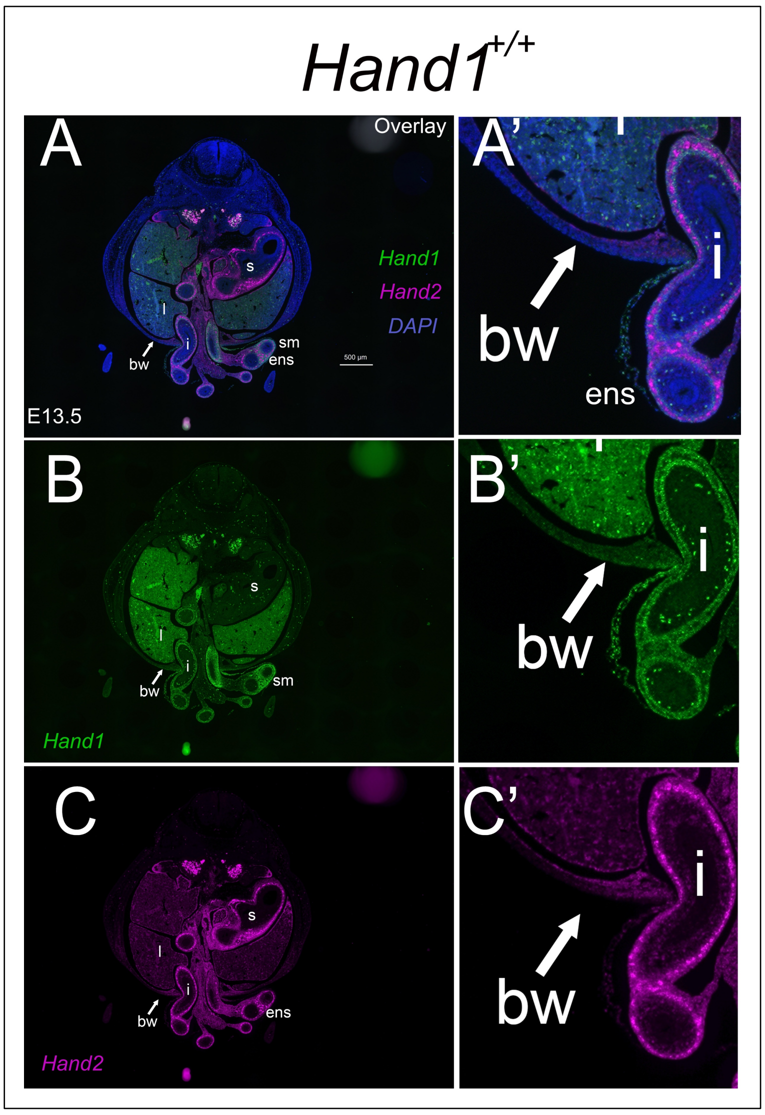

Fig. S4. RNAScope hybridization showing *Hand1* and *Hand2* expression within E13.5 transverse sections at the level of the umbilicus. A and A' overlay images of a wild type section showing *Hand1* (Green) and *Hand2* (magenta) with DAPI (blue) staining nuclei. *Hand1* expression appears at the most ventral aspect of the body wall (bw white arrow) where *Hand2* expression is more interior within the bw. **B and B'** single channel showing *Hand1* expression (white arrow marking the body wall expression). **C and C'** single channel showing *Hand2* expression (white arrow marking the body wall expression).

Development • Supplementary information

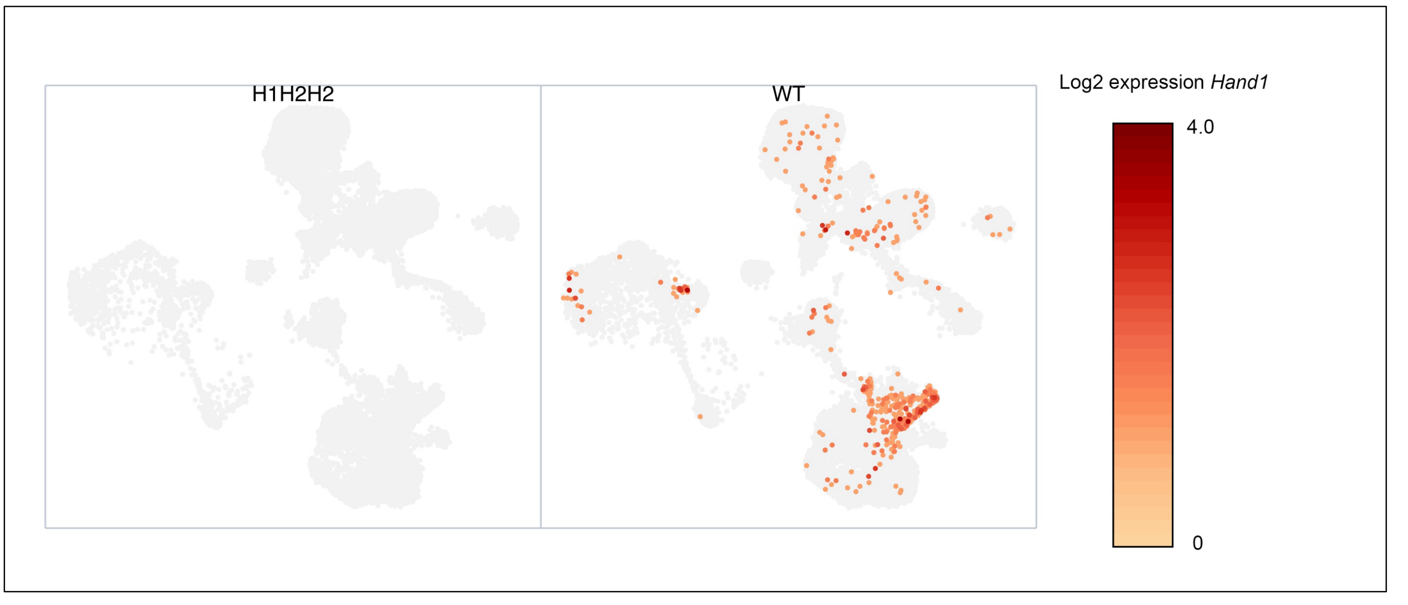

Fig. S5. UMAP gene feature representation of E13.5 single cell analysis from six control and six *Hand1*$^{Hand2/Hand2}$ embryo transverse sections. Gene features analysis of *Hand1* expression reveals that only a small percentage of cells express *Hand1* which hindered the identification of important gene expression changes.

Development • Supplementary information

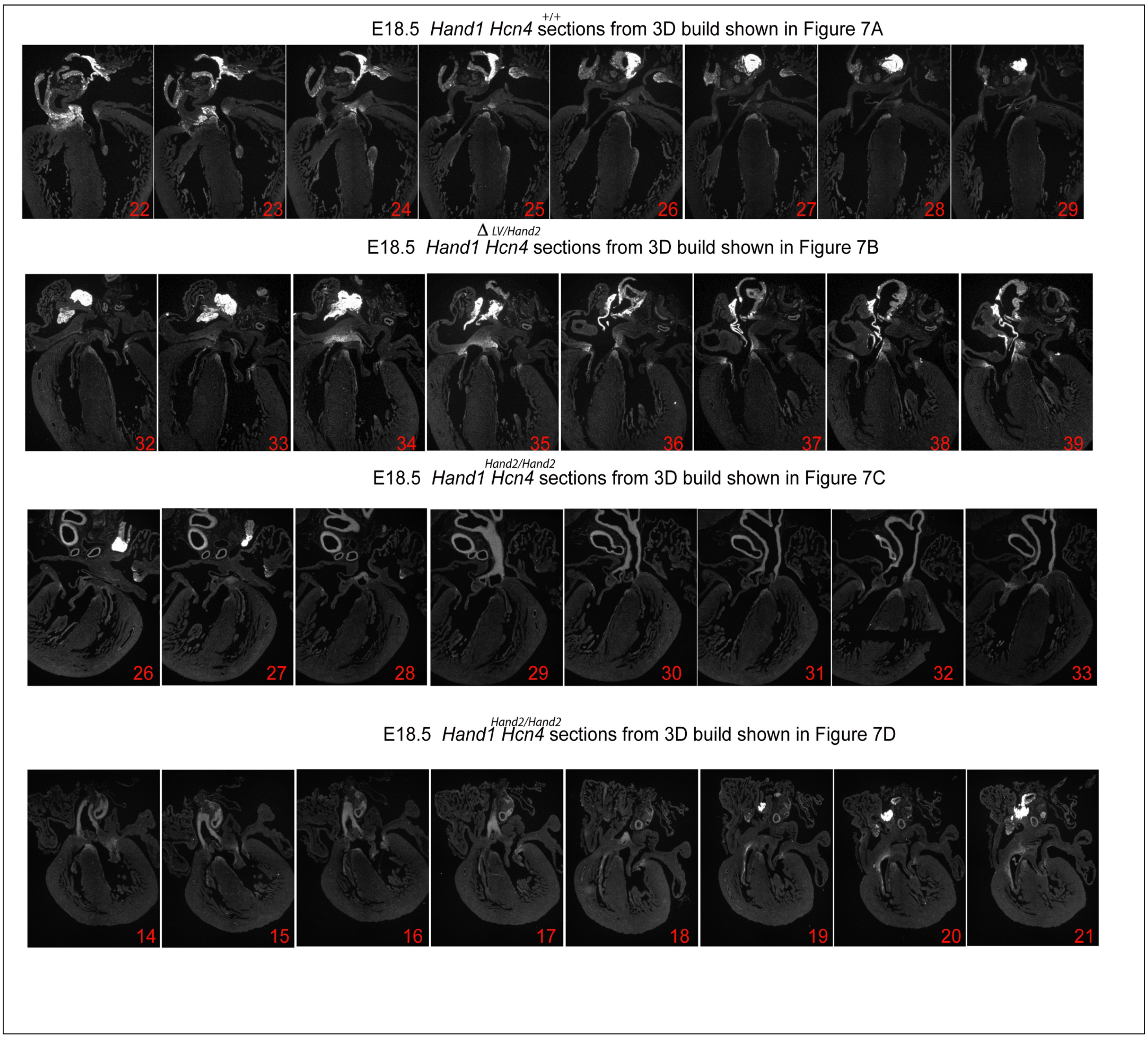

Fig. S6. Representative sections used for CCS 3D reconstructions shown in Fig. 8. Eight serial sections showing *Hcn4* expression are shown for each of the E18.5 examples that were 3D-reconstructed in Fig. 8. Section numbers are listed on each panel.

Table S1. Bulk Yolk sac RNA Seq differential expression spreadsheet.

Available for download at
https://journals.biologists.com/dev/article-lookup/doi/10.1242/dev.204963#supplementary-data

Table S2. IPA analysis *H1CKO* compared with *Wild type*.

Available for download at
https://journals.biologists.com/dev/article-lookup/doi/10.1242/dev.204963#supplementary-data

Table S3. IPA analysis *Hand1*$^{Hand2/Hand2}$ compared with *Wild type.*

Available for download at
https://journals.biologists.com/dev/article-lookup/doi/10.1242/dev.204963#supplementary-data

Table S4. IPA analysis *H1CKO* compared with *Hand1*$^{Hand2/Hand2}$.

Available for download at
https://journals.biologists.com/dev/article-lookup/doi/10.1242/dev.204963#supplementary-data

Table S5. Visium barcodes selected from Control sections.

Available for download at
https://journals.biologists.com/dev/article-lookup/doi/10.1242/dev.204963#supplementary-data

Table S6. Visium barcodes selected from *Hand1*$^{Hand2/Hand2}$ sections

Available for download at
https://journals.biologists.com/dev/article-lookup/doi/10.1242/dev.204963#supplementary-data

Table S7. Cluster ID marker expression

Available for download at
https://journals.biologists.com/dev/article-lookup/doi/10.1242/dev.204963#supplementary-data

Table S8. DE expression within individual Visium Clusters

Available for download at
https://journals.biologists.com/dev/article-lookup/doi/10.1242/dev.204963#supplementary-data

Table S9. list of mouse lines used in this study.

Allele	Reference #
Hand1^{LacZ}, *Hand1* systemic knockout allele	**Firulli, A. B., McFadden, D. G., Lin, Q., Srivastava, D. and Olson, E. N.** (1998). Heart and extra-embryonic mesodermal defects in mouse embryos lacking the bHLH transcription factor Hand1. *Nature Genetics* **18**, 266-270
Hand1^{fx}, *Hand1* conditional knockout allele	**McFadden, D. G., Barbosa, A. C., Richardson, J. A., Schneider, M. D., Srivastava, D. and Olson, E. N.** (2005). The Hand1 and Hand2 transcription factors regulate expansion of the embryonic cardiac ventricles in a gene dosage-dependent manner. *Development* **132**, 189-201.
T-Cre, Brachyury Cre Recombinase driver.	**Perantoni, A. O., Timofeeva, O., Naillat, F., Richman, C., Pajni-Underwood, S., Wilson, C., Vainio, S., Dove, L. F. and Lewandoski, M.** (2005). Inactivation of FGF8 in early mesoderm reveals an essential role in kidney development. *Development* **132**, 3859-3871.
Hand1$^{\Delta LV/\Delta LV}$, *Hand1* left ventricular deletion allele	**Vincentz, J. W., Firulli, B. A., Toolan, K. P., Arking, D. E., Sotoodehnia, N., Wan, J., Chen, P. S., de Gier-de Vries, C., Christoffels, V. M., Rubart-von der Lohe, M., et al.** (2019). Variation in a Left Ventricle-Specific Hand1 Enhancer Impairs GATA Transcription Factor Binding and Disrupts Conduction System Development and Function. *Circ Res* **125**, 575-589 doi 10.1161/CIRCRESAHA.119.315313
Hand1$^{Hand2/Hand2}$	This study Supplemental Figure 1

Development • Supplementary information
