# Supplementary material for: Hand1 gene replacement with Hand2 reveals overlap in function with unique occurrence of omphalocele and heart defects
Source: Development. 2025 Oct 14;152(19):dev204963. doi: 10.1242/dev.204963 (PMC12587295; doi:10.1242/dev.204963)
Supplement: Table S4. [file develop-152-204963-TableS4.zip › Firulli 2025 Supplemental File 4 IPA H1CKOvsH1H2H2.pdf]

INGENUITY
PATHWAY ANALYSIS

Analysis Name: H1.H2H2vsH1CKO
Analysis Creation Date: 2019-08-15
Build version: exported
Content version: 48207413 (Release Date: 2019-06-15)

Experiment Metadata

Name	Value

Analysis Settings

Reference set: Ingenuity Knowledge Base (Genes Only)
Relationship to include: Direct and Indirect
Does not Include Endogenous Chemicals
Optional Analyses: My Pathways My List

Filter Summary:
Consider only molecules and/or relationships where
(species = Mouse) AND
(tissues/cell lines = Small Intestine OR CNS Cell Lines not otherwise specified OR Mast cells OR Ovarian Cancer Cell Lines not otherwise specified OR Swiss 3T3 cells OR MDA-MB-468 OR Other Hepatoma Cell Lines OR Hippocampus OR Peripheral blood leukocytes not otherwise specified OR Other Monocytes OR Blood platelets OR Epithelial cells not otherwise specified OR Dorsal Root Ganglion OR Pro-B lymphocytes OR Fibroblast cell lines not otherwise specified OR MG-63 OR Heart OR Macrophages not otherwise specified OR Monocyte-derived

(c) 2000-2019 QIAGEN. All rights reserved.

macrophage OR Smooth Muscle OR MEF cells OR SK-MEL-5 OR Bladder OR Immature monocyte-derived dendritic cells OR Pancreatic Cancer Cell Lines not otherwise specified OR Other Kidney cell lines OR Other Granulocytes OR NCI-ADR-RES OR Brain OR IGROV1 OR Other Leukemia Cell Lines OR Other Teratocarcinoma Cell Lines OR BA/F3 OR Adrenal Gland OR Adipocytes OR MCF7 OR Th17 cells OR Thymus OR Kidney OR HOP-92 OR B lymphocytes not otherwise specified OR Colon Cancer Cell Lines not otherwise specified OR NT2/D1 OR K-562 OR CAKI-1 OR Other Neuroblastoma Cell Lines OR Other Macrophages OR 293 cells OR Other Fibroblast cell lines OR Caco2 cells OR EKVX OR MOLT-4 OR UACC-62 OR Gray Matter OR Naive B cells OR Calvaria OR Lung OR A375 OR CD4+ T-lymphocytes OR Cerebellum OR Lymphocytes not otherwise specified OR Teratocarcinoma Cell Lines not otherwise specified OR Hypothalamus OR Skin OR Immune cells not otherwise specified OR BT-549 OR Thyroid Gland OR BT-474 OR Cartilage Tissue OR Bone marrow cells not otherwise specified OR HEL OR SN12C OR Trachea OR NCI-H522 OR 786-0 OR Testis OR Other NK cells OR BDCA-1+ dendritic cells OR Putamen OR RBL-2H3 OR Cortical neurons OR Cell Line not otherwise specified OR Hepatoma Cell Lines not otherwise specified OR MDA-MB-231 OR Other Lymphoma Cell Lines OR LNCaP cells OR Pituitary Gland OR Other Kidney Cancer Cell Lines OR J-774A.1 OR PC-12 cells OR Epidermis OR Bone marrow-derived macrophages OR Nucleus Accumbens OR Oocytes OR Vascular smooth muscle cells OR Other Memory T lymphocytes OR Other Endothelial cells OR T47-D OR Cos-7 cells OR NCI-H332M OR HMC-1 OR UO-31 OR Osteoblasts OR U87MG OR Memory B cells OR Megakaryocytes OR Purkinje cells OR Langerhans cells OR Other Cervical cancer cell line OR Caudate Nucleus OR SF-539 OR Pyramidal neurons OR Immune cell lines not otherwise specified OR MDA-N OR Effector memory RA+ cytotoxic T cells OR Trigeminal Ganglion OR Natural T-regulatory cells OR Other Stem cells OR PBMCs OR Kidney Cancer Cell Lines not otherwise specified OR KM-12 OR CCRF-CEM OR Other Mononuclear leukocytes OR Dermis OR Myeloid dendritic cells OR Cornea OR Other Melanoma Cell Lines OR Substantia Nigra OR Granulocytes not otherwise specified OR Corpus Callosum OR CD34+ cells OR Other Dendritic cells OR Pancreas OR Effector T cells OR Lymphoma Cell Lines not otherwise specified OR A549-ATCC OR Kidney cell lines not otherwise specified OR NB4 OR Large Intestine OR INS-1 OR Th1 cells OR Plasma cells OR Forestomach OR Microglia OR HeLa OR Other Immune cells OR Neuroblastoma Cell Lines not otherwise specified OR Mesenchymal stem cells OR PC-3 OR Other Organ Systems OR Fibroblasts OR Crypt OR Cervical cancer cell line not otherwise specified OR HOP-62 OR Placenta OR Adipose OR Cerebral Ventricles OR Peripheral blood lymphocytes OR Cerebral Cortex OR HepG2 OR Cardiomyocytes OR SW-480 OR Amygdala OR Cells not otherwise specified OR Peripheral blood monocytes OR Subventricular Zone OR CD56dim NK cells OR Spleen OR HuH7 OR SK-MEL-28 OR Activated helper T cells OR Macrophage Cancer Cell Lines not otherwise specified OR Effector memory helper T cells OR Activated Vd1 Gamma-delta T cells OR PANC-1 OR Endothelial cells not otherwise specified OR MDA-MB-361 OR Effector memory cytotoxic T cells OR HCT-116 OR Salivary Gland OR Esophagus OR Monocytes not otherwise specified OR Breast Cancer Cell Lines not otherwise specified OR Other Myeloma Cell Lines OR U2OS OR P19 OR Keratinocytes OR Peritoneal macrophages OR Activated CD56bright NK cells OR NCI-H226 OR Striatum OR Thalamus OR Dendritic cells not otherwise specified OR Other Nervous System OR Other Pancreatic Cancer Cell Lines OR OVCAR-5 OR Ovary OR Activated CD56dim NK cells OR Other Prostate Cancer

(c) 2000-2019 QIAGEN. All rights reserved.

Cell Lines OR Other Neurons OR Stromal cells OR SW-620 OR Neutrophils OR Stomach OR Other Tissues and Primary Cells OR Th2 cells OR Vd2 Gamma-delta T cells OR OVCAR-3 OR Mature monocyte-derived dendritic cells OR Microvascular endothelial cells OR Other Ovarian Cancer Cell Lines OR 3T3-L1 cells OR Murine NKT cells OR RKO OR Plasmacytoid dendritic cells OR SF-295 OR HCT-15 OR SK-N-SH OR Osteosarcoma Cell Lines not otherwise specified OR Retina OR Other B lymphocytes OR Myeloma Cell Lines not otherwise specified OR Other Lymphocytes OR Lymph node OR A2780 OR Pre-B lymphocytes OR MALME-3M OR UACC-257 OR Min6 OR Pheochromocytoma cell lines not otherwise specified OR Central memory helper T cells OR Olfactory Bulb OR H460 OR SR OR Other Epithelial cells OR Prostate Gland OR Other Lung Cancer Cell Lines OR Vd1 Gamma-delta T cells OR Memory T lymphocytes not otherwise specified OR Other Smooth muscle cells OR NCI-H23 OR Naive helper T cells OR Leukemia Cell Lines not otherwise specified OR Brainstem OR Other Cell Line OR Granulosa cells OR Sertoli cells OR T lymphocytes not otherwise specified OR Thymocytes OR Stem cells not otherwise specified OR Hep3B OR Other Immune cell lines OR Prostate Cancer Cell Lines not otherwise specified OR Other Macrophage Cancer Cell Lines OR Other Pheochromocytoma cell lines OR HT29 OR Granule cells OR Melanocytes OR Choroid Plexus OR SF-268 OR Nervous System not otherwise specified OR Splenocytes OR HCC-2998 OR CD56bright NK cells OR Other T lymphocytes OR RXF-393 OR Monocyte-derived dendritic cells not otherwise specified OR Embryonic stem cells OR HL-60 OR U251 OR HUVEC cells OR Uterus OR RPMI-8266 OR U937 OR Medulla Oblongata OR Lung Cancer Cell Lines not otherwise specified OR Cytotoxic T cells OR SNB-75 OR Beta islet cells OR OVCAR-4 OR NK cells not otherwise specified OR TK-10 OR Parietal Lobe OR Eosinophils OR OVCAR-8 OR U266 OR Spinal Cord OR HS 578T OR Activated Vd2 Gamma-delta T cells OR Mammary Gland OR WEHI-231 OR ACHN OR COLO205 OR LOX IMVI OR Intraepithelial T lymphocytes OR Tissues and Primary Cells not otherwise specified OR DU-145 OR Granule Cell Layer OR Other Breast Cancer Cell Lines OR THP-1 OR Ventricular Zone OR RAW 264.7 OR Hepatocytes OR Hematopoietic progenitor cells OR M14 OR Liver OR Other Colon Cancer Cell Lines OR Mononuclear leukocytes not otherwise specified OR Other Osteosarcoma Cell Lines OR Organ Systems not otherwise specified OR SK-OV-3 OR Jurkat OR Bone marrow-derived dendritic cells OR Central memory cytotoxic T cells OR Other CNS Cell Lines OR Lens OR Other Bone marrow cells OR Skeletal Muscle OR White Matter OR Other Monocyte-derived dendritic cells OR Astrocytes OR Other Peripheral blood leukocytes OR Sciatic Nerve OR MDA-MB-435 OR BDCA-3+ dendritic cells OR Smooth muscle cells not otherwise specified OR A498 OR Neurons not otherwise specified OR J774 OR Other Cells OR SK-MEL-2 OR NIH/3T3 cells OR Chondrocytes OR Melanoma Cell Lines not otherwise specified) AND
(data sources = An Open Access Database of Genome-wide Association Results OR BIND OR BioGRID OR Catalogue Of Somatic Mutations In Cancer (COSMIC) OR Chemical Carcinogenesis Research Information System (CCRIS) OR ClinicalTrials.gov OR ClinVar OR Cognia OR DIP OR DrugBank OR Gene Ontology (GO) OR GVK Biosciences OR Hazardous Substances Data Bank (HSDB) OR HumanCyc OR Ingenuity Expert Findings OR Ingenuity ExpertAssist Findings OR IntAct OR Interactome studies OR MIPS OR miRBase OR miRecords OR Mouse Genome Database (MGD) OR Obesity Gene Map Database OR Online Mendelian Inheritance in Man (OMIM) OR TarBase OR TargetScan Human)

(c) 2000-2019 QIAGEN. All rights reserved.

Top Canonical Pathways

Name	p-value	Overlap
**Axonal Guidance Signaling**	1.02E-14	47.3 % 222/469
**Synaptogenesis Signaling Pathway**	5.10E-14	51.2 % 154/301
**Calcium Signaling**	2.66E-11	53.3 % 104/195
**Basal Cell Carcinoma Signaling**	1.40E-09	65.7 % 46/70
**Opioid Signaling Pathway**	1.52E-08	47.7 % 115/241

Top Upstream Regulators

Name	p-value	Predicted Activation
**TP53**	4.92E-15	Inhibited
**CTNNB1**	2.74E-11	Inhibited
**HNF1A**	2.93E-11	Activated
**HNF4A**	1.02E-09	Activated
**KDM5A**	6.36E-09	Inhibited

Top Diseases and Bio Functions

Diseases and Disorders

(c) 2000-2019 QIAGEN. All rights reserved.

INGENUITY PATHWAY ANALYSIS

Name	p-value range	# Molecules
Organismal Injury and Abnormalities	4.18E-05 - 3.50E-34	1492
Neurological Disease	3.29E-05 - 2.22E-29	950
Connective Tissue Disorders	4.18E-05 - 9.70E-15	329
Skeletal and Muscular Disorders	4.18E-05 - 9.70E-15	601
Auditory Disease	3.11E-05 - 1.28E-14	119

Molecular and Cellular Functions

Name	p-value range	# Molecules
Cellular Assembly and Organization	4.20E-05 - 2.37E-24	931
Cellular Function and Maintenance	4.14E-05 - 2.37E-24	1201
Cellular Development	3.83E-05 - 2.63E-24	762
Cellular Growth and Proliferation	2.31E-05 - 2.63E-24	542
Cell Morphology	3.62E-05 - 8.01E-21	841

Physiological System Development and Function

Name	p-value range	# Molecules
Organismal Development	4.29E-05 - 2.73E-35	2011
Embryonic Development	4.29E-05 - 6.29E-34	1304
Organismal Survival	8.72E-26 - 1.00E-32	1351
Nervous System Development and Function	3.49E-05 - 1.78E-31	1357
Organ Morphology	4.18E-05 - 4.68E-27	1151

(c) 2000-2019 QIAGEN. All rights reserved.

INGENUITY PATHWAY ANALYSIS

Top Tox Functions

Assays: Clinical Chemistry and Hematology

Name	p-value range	# Molecules
Increased Levels of Alkaline Phosphatase	3.06E-01 - 1.04E-02	25
Decreased Levels of Potassium	1.39E-01 - 1.39E-01	11
Increased Levels of Red Blood Cells	2.23E-01 - 1.75E-01	37
Decreased Levels of Hematocrit	2.05E-01 - 2.05E-01	4
Decreased Levels of Albumin	5.18E-01 - 2.23E-01	3

Cardiotoxicity

Name	p-value range	# Molecules
Congenital Heart Anomaly	6.65E-01 - 1.59E-11	114
Cardiac Enlargement	6.65E-01 - 2.31E-05	198
Cardiac Hypoplasia	3.06E-01 - 3.32E-04	33
Cardiac Stenosis	5.18E-01 - 7.10E-03	12
Cardiac Arteriopathy	3.58E-01 - 8.73E-03	10

Hepatotoxicity

Name	p-value range	# Molecules
Liver Damage	3.06E-01 - 1.69E-04	69

(c) 2000-2019 QIAGEN. All rights reserved.

INGENUITY PATHWAY ANALYSIS

**Liver Proliferation**	5.18E-01 - 3.25E-03	65
**Liver Necrosis/Cell Death**	6.65E-01 - 3.49E-03	95
**Liver Enlargement**	3.09E-02 - 3.09E-02	37
**Liver Regeneration**	3.58E-01 - 4.85E-02	22

Nephrotoxicity

Name	p-value range	# Molecules
**Renal Hypoplasia**	5.18E-01 - 3.06E-05	38
**Glomerular Injury**	1.00E00 - 2.19E-03	119
**Kidney Failure**	3.06E-01 - 2.19E-03	44
**Renal Fibrosis**	1.00E00 - 2.19E-03	34
**Renal Damage**	5.18E-01 - 7.92E-03	54

Top Regulator Effect Networks

ID	Regulators	Disease & Functions	Consistency Score
**1**	EED,FGF9,TGFBR1	Aplasia,Cell movement of neurons (+8 more)	15.119
**2**	CHRD,DLL3,FOXC1,FOXC2,GATA4,MIB1,MSGN1,Pde 4,PDE8A (+1 more)	Cell-cell contact,Development of body trunk (+8 more)	12.09
**3**	DLL3,EED,FGF9,MIB1,MSGN1,TGFBR1	Development of genitourinary system (+3 more)	12.07
**4**	COMMD3-BMI1,KAT6A,SRF	Aplasia,Fusion of cervical vertebra (+6 more)	11.854
**5**	COMMD3-BMI1,Hdac,KAT6A,KMT2A,MIR17HG,SHH	Branching morphogenesis of metanephric bud (+12 more)	10.854

Top Networks

(c) 2000-2019 QIAGEN. All rights reserved.

INGENUITY PATHWAY ANALYSIS

ID	Associated Network Functions	Score
1	Lipid Metabolism, Small Molecule Biochemistry, Drug Metabolism	17
2	Connective Tissue Development and Function, Connective Tissue Disorders, Nervous System Development and Function	17
3	Skeletal and Muscular System Development and Function, Cellular Assembly and Organization, Protein Synthesis	17
4	Cellular Development, Embryonic Development, Nervous System Development and Function	17
5	Cell Death and Survival, Cardiac Arrythmia, Cardiovascular Disease	17

Top Tox Lists

Name	p-value	Overlap

(c) 2000-2019 QIAGEN. All rights reserved.

INGENUITY PATHWAY ANALYSIS

Renal Necrosis/Cell Death	5.03E-07	40.3 % 223/553
FXR/RXR Activation	1.51E-05	49.6 % 57/115
LPS/IL-1 Mediated Inhibition of RXR Function	3.96E-05	43.0 % 99/230
LXR/RXR Activation	1.42E-04	47.3 % 53/112
Cardiac Hypertrophy	1.07E-03	38.2 % 139/364

Top My Lists

Name	p-value	Overlap
Q6 B	2.82E-14	35.8 % 1307/3652
q7	3.11E-14	35.8 % 1307/3653
LoG v LoC common -+gender from VenD 10 -25-19	4.77E-12	42.5 % 303/713
combG v LoC -+gender common from VenD 10-25-18	2.79E-11	39.8 % 425/1068
chondrogensis	3.47E-11	62.1 % 64/103

Top My Pathways

Name	p-value	Overlap
Lipid M-2	1.33E-04	69.6 % 16/23
New My Pathway ipa exam q4	4.31E-04	51.6 % 32/62
IPA 21015	4.31E-04	51.6 % 32/62
TNBC/basal like genes	2.42E-03	41.1 % 69/168
GNASdelme	2.05E-02	52.0 % 13/25

(c) 2000-2019 QIAGEN. All rights reserved.

INGENUITY PATHWAY ANALYSIS

Top Analysis-Ready Molecules

Expr Fold Change

Molecules	Expr. Value	Chart
**GPR55**	↑ 131.566	
**Cldn34c1 (includes others)**	↑ 93.593	
**NEU2**	↑ 87.778	
**IFI16**	↑ 44.555	
**ALOX12B**	↑ 27.744	
**Gstm6**	↑ 24.472	
**TMEM252**	↑ 24.192	
**Btnl4/Btnl6**	↑ 20.997	
**AOAH**	↑ 17.124	
**Ces2e**	↑ 16.144	

Expr Fold Change

Molecules	Expr. Value	Chart

(c) 2000-2019 QIAGEN. All rights reserved.

INGENUITY PATHWAY ANALYSIS

**MYH7**	↓	-5312.213
**SIX1**	↓	-1888.153
**FSD2**	↓	-1426.415
**MYL2**	↓	-1383.703
**OTX2**	↓	-1233.920
**MYH6**	↓	-1133.900
**5430431A17Rik**	↓	-1061.352
**FOXB1**	↓	-1012.768
**Miat**	↓	-963.706
**SOX1**	↓	-915.353

(c) 2000-2019 QIAGEN. All rights reserved.

INGENUITY PATHWAY ANALYSIS
